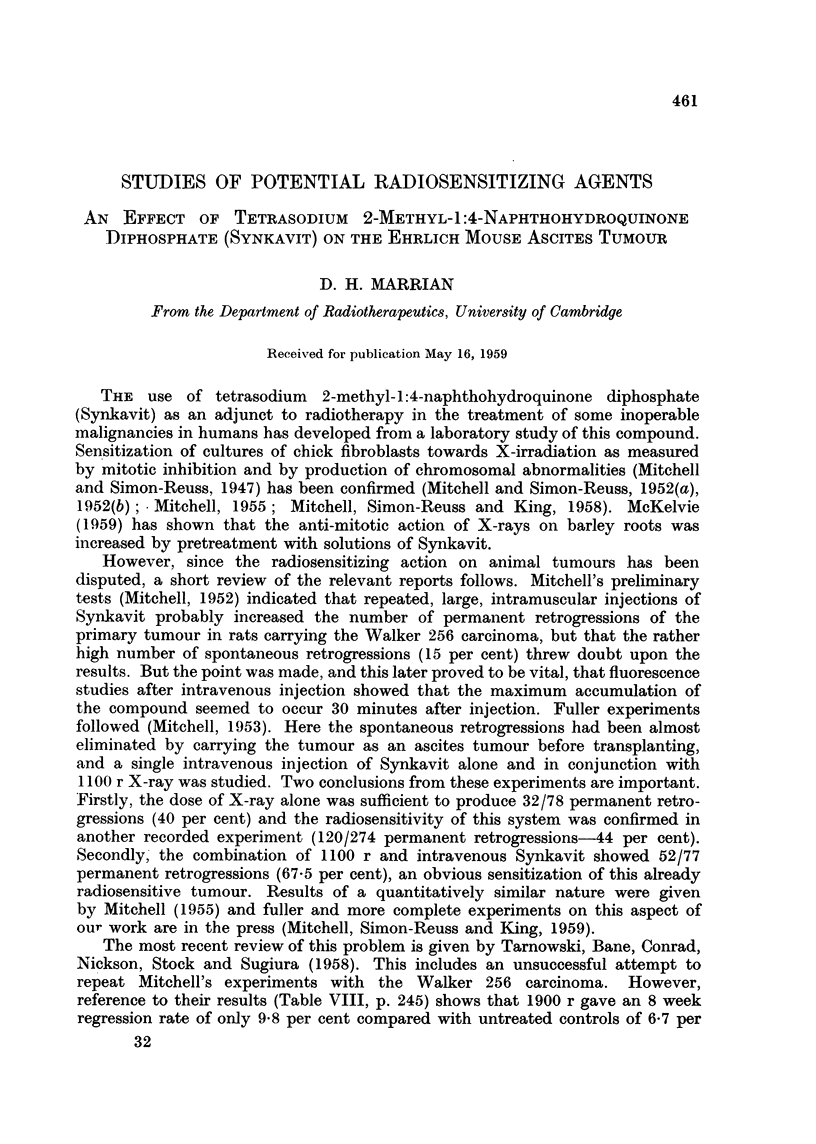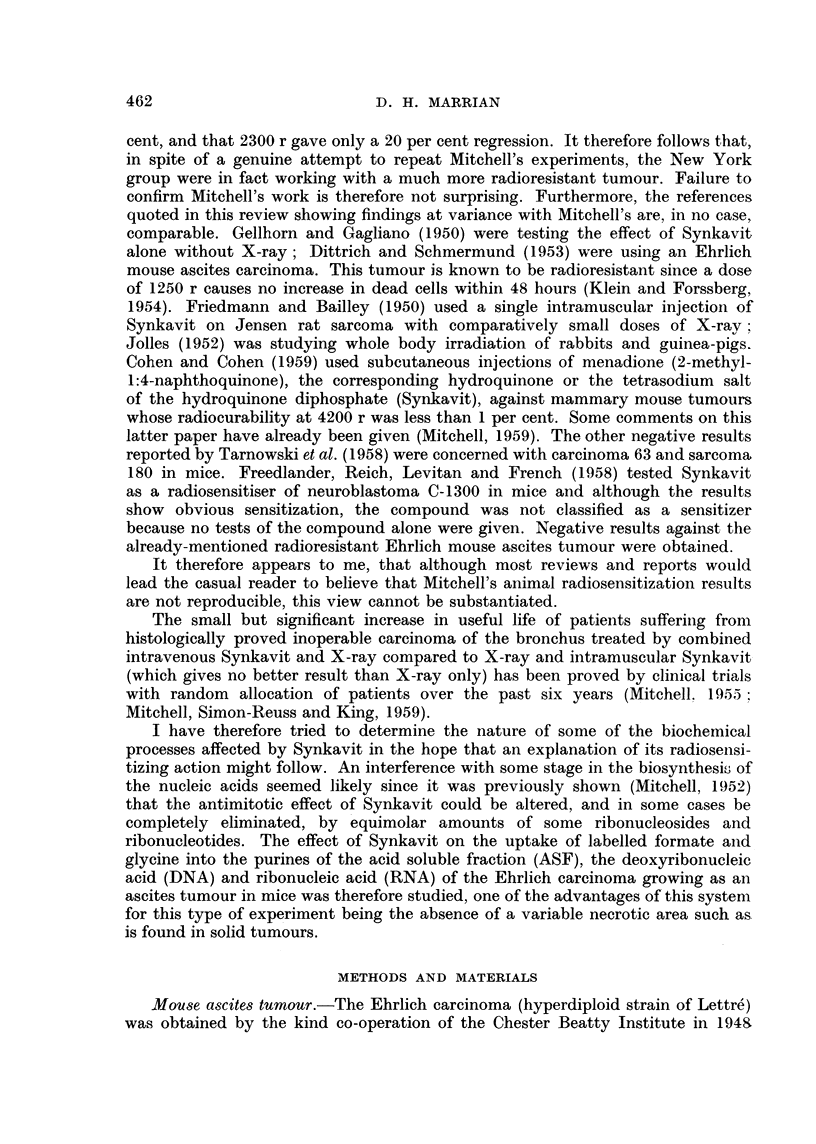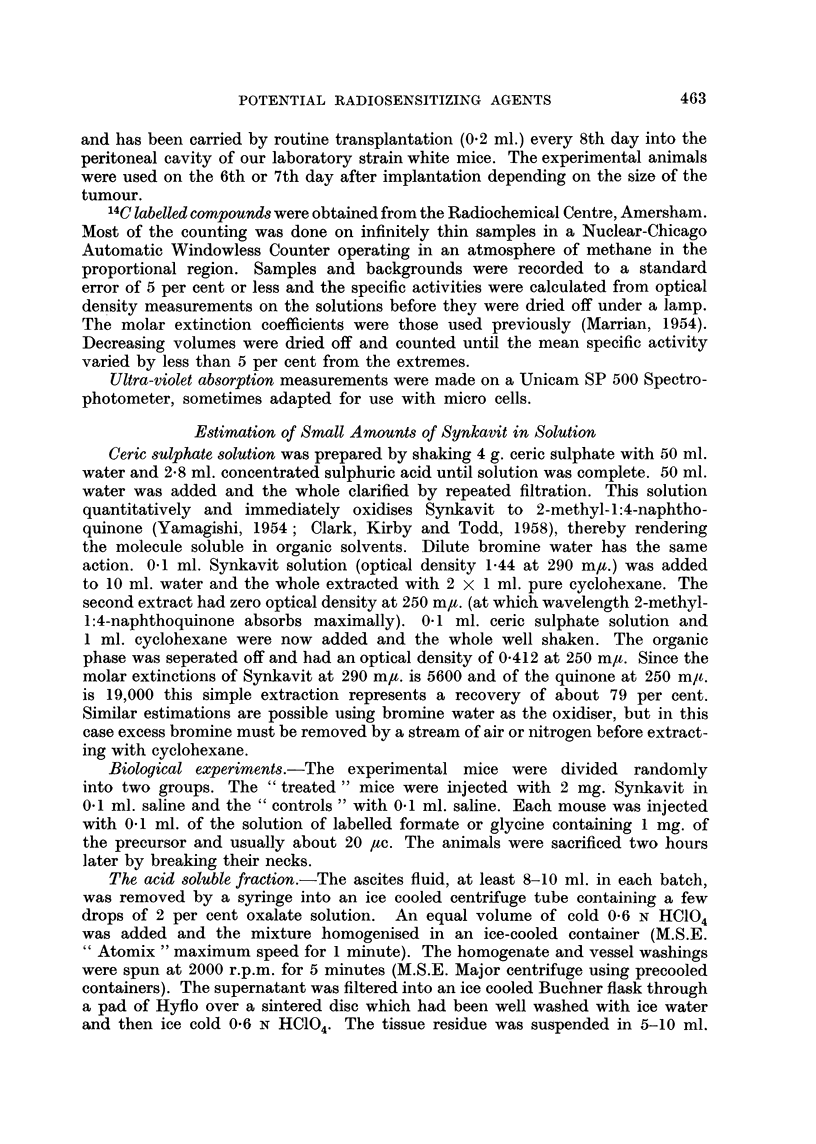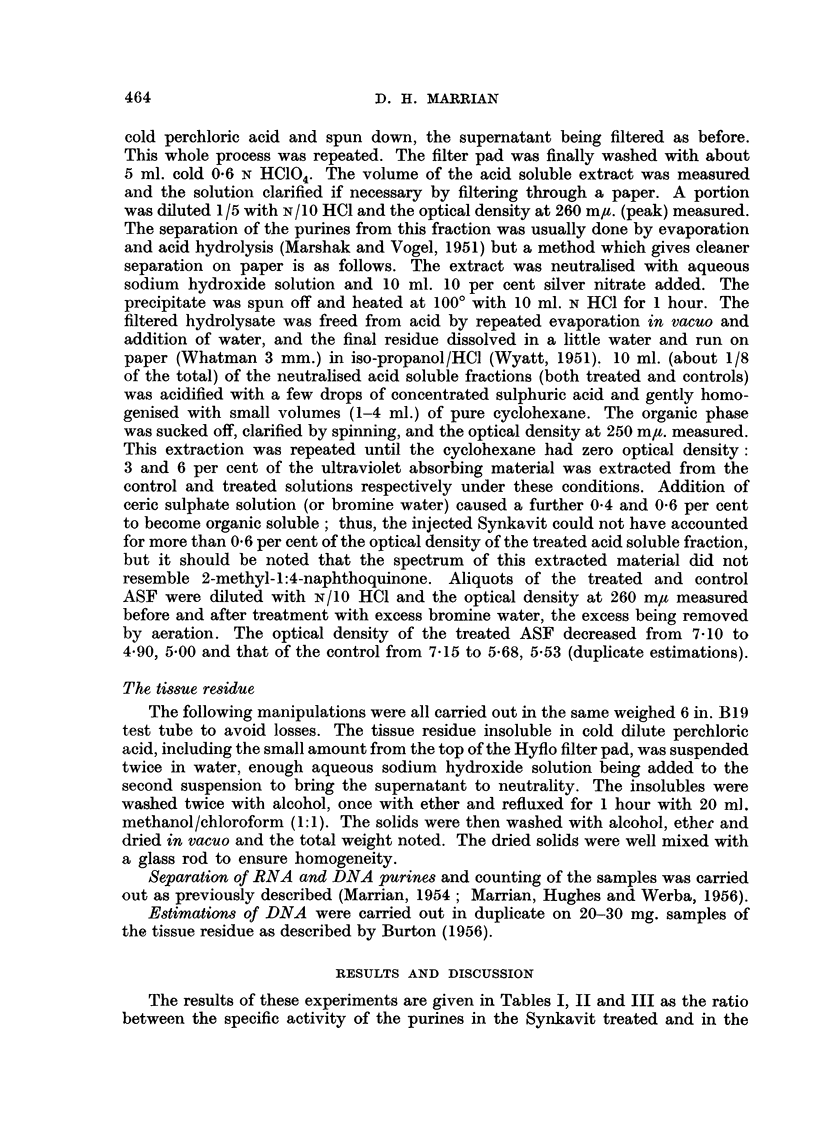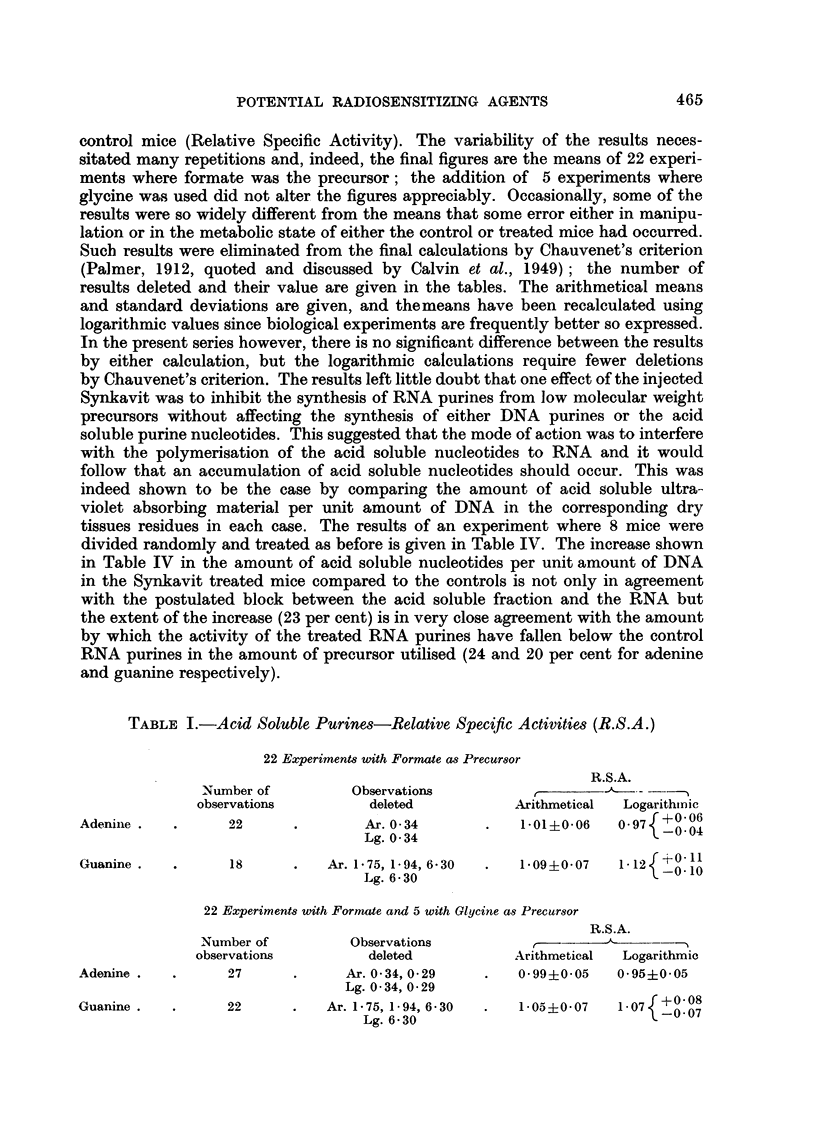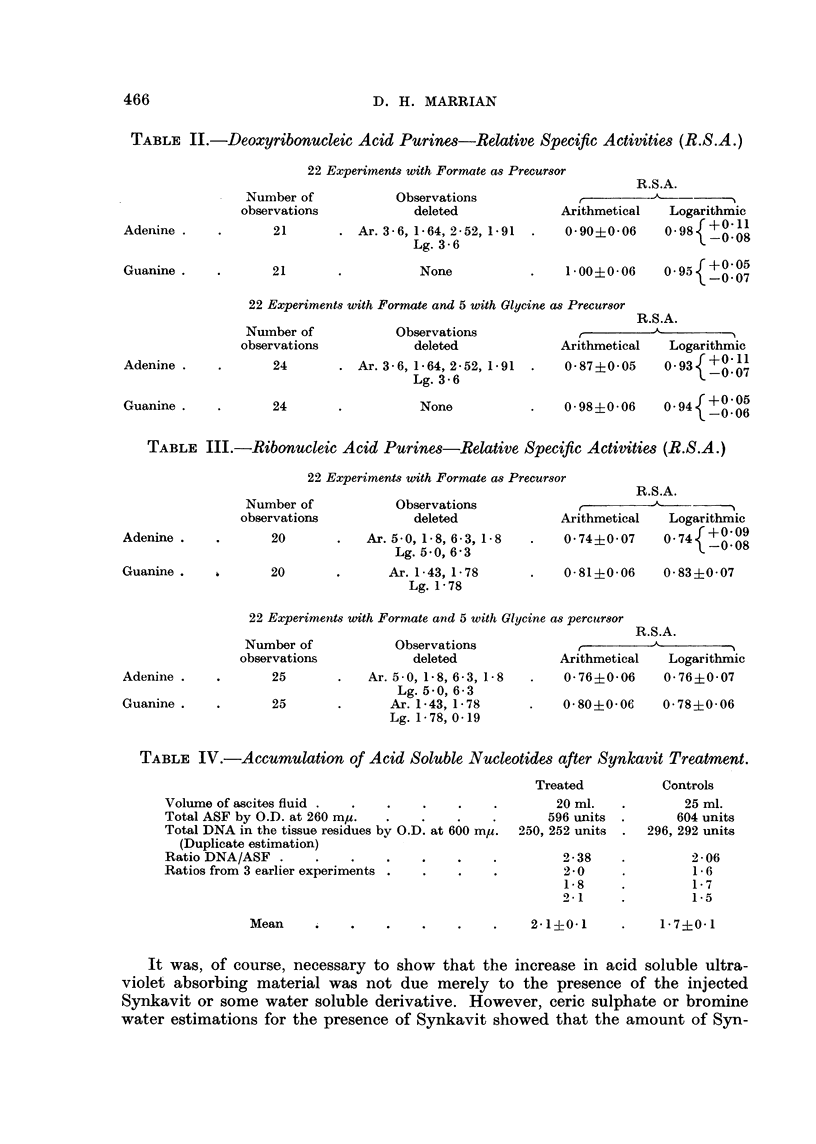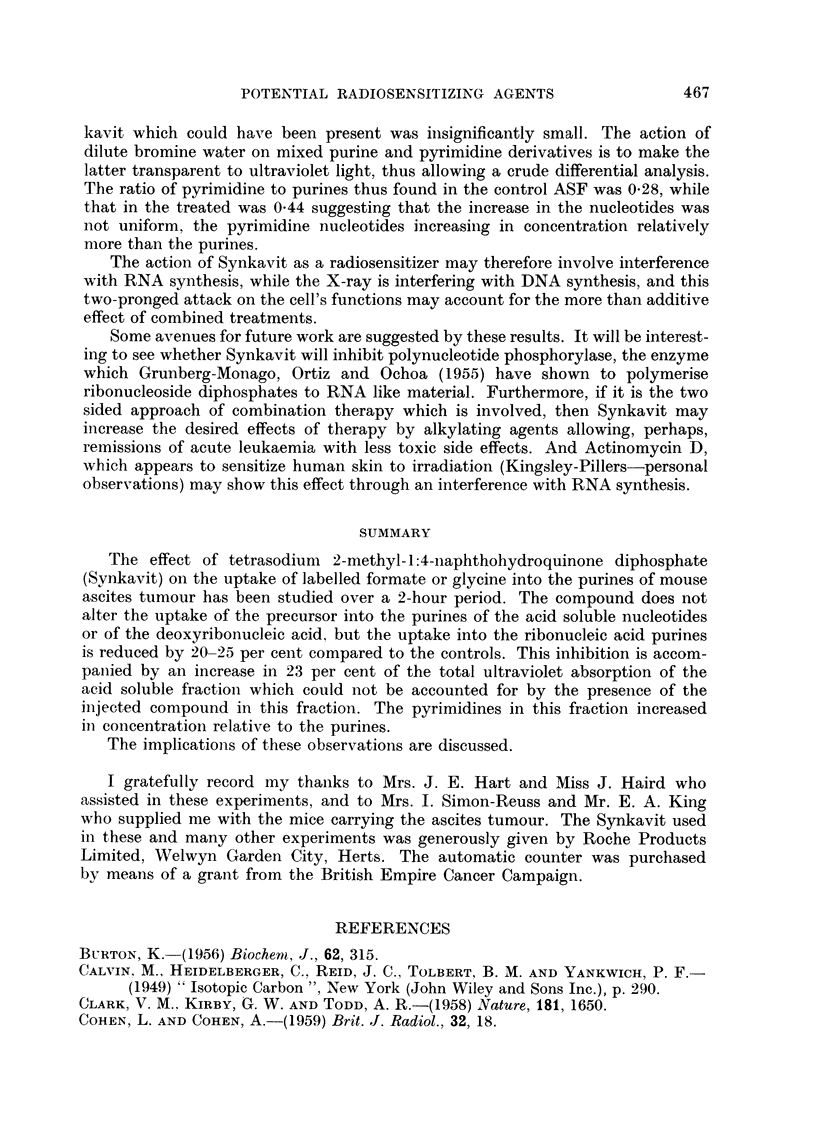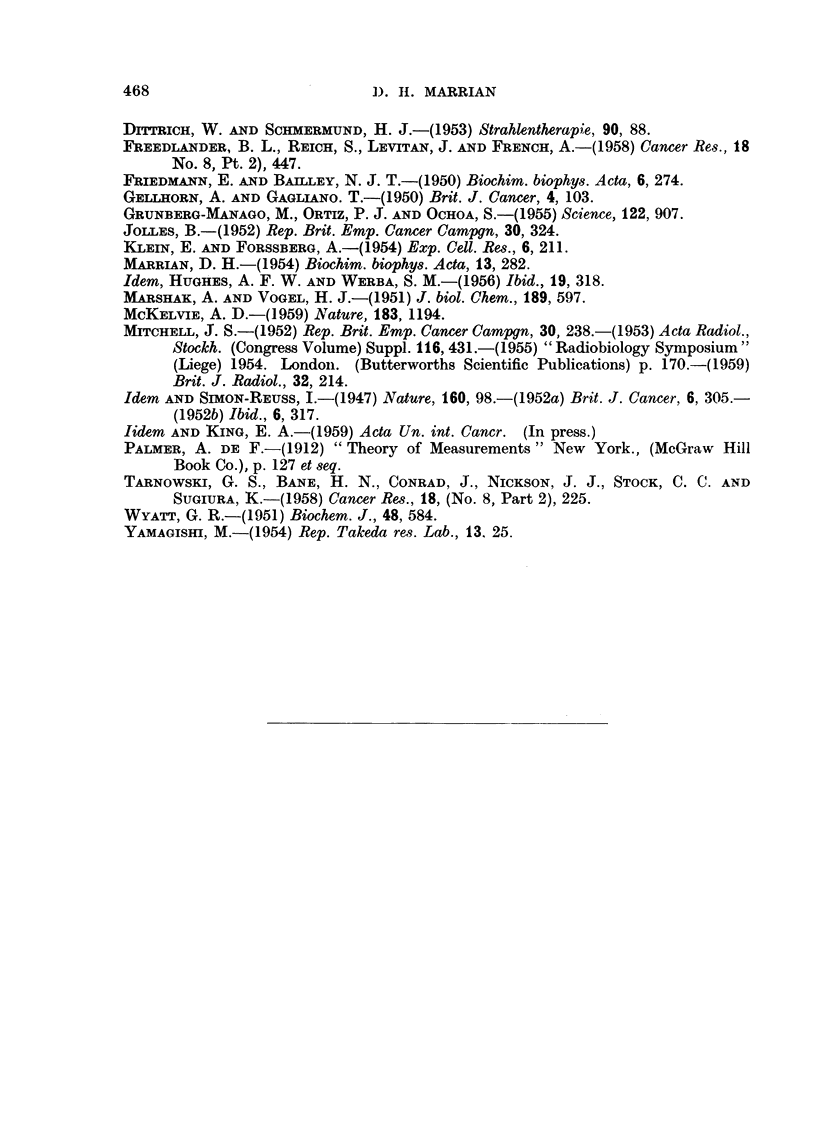# Studies of Potential Radiosensitizing Agents

**DOI:** 10.1038/bjc.1959.51

**Published:** 1959-09

**Authors:** D. H. Marrian


### STUDIES OF POTENTIAL RADIOSENSITIZING AGENTS

### An Effect of Tetrasodium 2-Methyl-1:4-Naphthohydroquinone Diphosphate (Synkavit) on the Ehrlich Mouse Ascites Tumour

D. H. MARRIAN

*From the Department of Radiotherapeutics, University of Cambridge*

Received for publication May 16, 1959

The use of tetrasodium 2-methyl-1:4-naphthohydroquinone diphosphate (Synkavit) as an adjunct to radiotherapy in the treatment of some inoperable malignancies in humans has developed from a laboratory study of this compound. Sensitization of cultures of chick fibroblasts towards X-irradiation as measured by mitotic inhibition and by production of chromosomal abnormalities (Mitchell and Simon-Reuss, 1947) has been confirmed (Mitchell and Simon-Reuss, 1952(*a*), 1952(*b*); Mitchell, 1955; Mitchell, Simon-Reuss and King, 1958). McKelvie (1959) has shown that the anti-mitotic action of X-rays on barley roots was increased by pretreatment with solutions of Synkavit.

However, since the radiosensitizing action on animal tumours has been disputed, a short review of the relevant reports follows. Mitchell's preliminary tests (Mitchell, 1952) indicated that repeated, large, intramuscular injections of Synkavit probably increased the number of permanent retrogressions of the primary tumour in rats carrying the Walker 256 carcinoma, but that the rather high number of spontaneous retrogressions (15 per cent) threw doubt upon the results. But the point was made, and this later proved to be vital, that fluorescence studies after intravenous injection showed that the maximum accumulation of the compound seemed to occur 30 minutes after injection. Fuller experiments followed (Mitchell, 1953). Here the spontaneous retrogressions had been almost eliminated by carrying the tumour as an ascites tumour before transplanting, and a single intravenous injection of Synkavit alone and in conjunction with 1100 r X-ray was studied. Two conclusions from these experiments are important. Firstly, the dose of X-ray alone was sufficient to produce 32/78 permanent retrogressions (40 per cent) and the radiosensitivity of this system was confirmed in another recorded experiment (120/274 permanent retrogressions—44 per cent). Secondly, the combination of 1100 r and intravenous Synkavit showed 52/77 permanent retrogressions (67·5 per cent), an obvious sensitization of this already radiosensitive tumour. Results of a quantitatively similar nature were given by Mitchell (1955) and fuller and more complete experiments on this aspect of our work are in the press (Mitchell, Simon-Reuss and King, 1959).

The most recent review of this problem is given by Tarnowski, Bane, Conrad, Nickson, Stock and Sugiura (1958). This includes an unsuccessful attempt to repeat Mitchell's experiments with the Walker 256 carcinoma. However, reference to their results (Table VIII, p. 245) shows that 1900 r gave an 8 week regression rate of only 9·8 per cent compared with untreated controls of 6·7 per cent, and that 2300 r gave only a 20 per cent regression. It therefore follows that, in spite of a genuine attempt to repeat Mitchell's experiments, the New York group were in fact working with a much more radioresistant tumour. Failure to confirm Mitchell's work is therefore not surprising. Furthermore, the references quoted in this review showing findings at variance with Mitchell's are, in no case, comparable. Gellhorn and Gagliano (1950) were testing the effect of Synkavit alone without X-ray ; Dittrich and Schmermund (1953) were using an Ehrlich mouse ascites carcinoma. This tumour is known to be radioresistant since a dose of 1250 r causes no increase in dead cells within 48 hours (Klein and Forssberg, 1954). Friedmann and Bailley (1950) used a single intramuscular injection of Synkavit on Jensen rat sarcoma with comparatively small doses of X-ray ; Jolles (1952) was studying whole body irradiation of rabbits and guinea-pigs. Cohen and Cohen (1959) used subcutaneous injections of menadione (2-methyl-1:4-naphthoquinone), the corresponding hydroquinone or the tetrasodium salt of the hydroquinone diphosphate (Synkavit), against mammary mouse tumours whose radiocurability at 4200 r was less than 1 per cent. Some comments on this latter paper have already been given (Mitchell, 1959). The other negative results reported by Tarnowski *et al.* (1958) were concerned with carcinoma 63 and sarcoma 180 in mice. Freedlander, Reich, Levitan and French (1958) tested Synkavit as a radiosensitiser of neuroblastoma C-1300 in mice and although the results show obvious sensitization, the compound was not classified as a sensitizer because no tests of the compound alone were given. Negative results against the already-mentioned radioresistant Ehrlich mouse ascites tumour were obtained.

It therefore appears to me, that although most reviews and reports would lead the casual reader to believe that Mitchell's animal radiosensitization results are not reproducible, this view cannot be substantiated.

The small but significant increase in useful life of patients suffering from histologically proved inoperable carcinoma of the bronchus treated by combined intravenous Synkavit and X-ray compared to X-ray and intramuscular Synkavit (which gives no better result than X-ray only) has been proved by clinical trials with random allocation of patients over the past six years (Mitchell, 1955 ; Mitchell, Simon-Reuss and King, 1959).

I have therefore tried to determine the nature of some of the biochemical processes affected by Synkavit in the hope that an explanation of its radiosensitizing action might follow. An interference with some stage in the biosynthesis of the nucleic acids seemed likely since it was previously shown (Mitchell, 1952) that the antimitotic effect of Synkavit could be altered, and in some cases be completely eliminated, by equimolar amounts of some ribonucleosides and ribonucleotides. The effect of Synkavit on the uptake of labelled formate and glycine into the purines of the acid soluble fraction (ASF), the deoxyribonucleic acid (DNA) and ribonucleic acid (RNA) of the Ehrlich carcinoma growing as an ascites tumour in mice was therefore studied, one of the advantages of this system for this type of experiment being the absence of a variable necrotic area such as is found in solid tumours.

### METHODS AND MATERIALS

*Mouse ascites tumour.*—The Ehrlich carcinoma (hyperdiploid strain of Lettré) was obtained by the kind co-operation of the Chester Beatty Institute in 1948

and has been carried by routine transplantation (0·2 ml.) every 8th day into the peritoneal cavity of our laboratory strain white mice. The experimental animals were used on the 6th or 7th day after implantation depending on the size of the tumour.

*$^{14}C$ labelled compounds* were obtained from the Radiochemical Centre, Amersham. Most of the counting was done on infinitely thin samples in a Nuclear-Chicago Automatic Windowless Counter operating in an atmosphere of methane in the proportional region. Samples and backgrounds were recorded to a standard error of 5 per cent or less and the specific activities were calculated from optical density measurements on the solutions before they were dried off under a lamp. The molar extinction coefficients were those used previously (Marrian, 1954). Decreasing volumes were dried off and counted until the mean specific activity varied by less than 5 per cent from the extremes.

*Ultra-violet absorption* measurements were made on a Unicam SP 500 Spectrophotometer, sometimes adapted for use with micro cells.

### *Estimation of Small Amounts of Synkavit in Solution*

*Ceric sulphate solution* was prepared by shaking 4 g. ceric sulphate with 50 ml. water and 2·8 ml. concentrated sulphuric acid until solution was complete. 50 ml. water was added and the whole clarified by repeated filtration. This solution quantitatively and immediately oxidises Synkavit to 2-methyl-1:4-naphthoquinone (Yamagishi, 1954; Clark, Kirby and Todd, 1958), thereby rendering the molecule soluble in organic solvents. Dilute bromine water has the same action. 0·1 ml. Synkavit solution (optical density 1·44 at 290 m$\mu$.) was added to 10 ml. water and the whole extracted with 2 $\times$ 1 ml. pure cyclohexane. The second extract had zero optical density at 250 m$\mu$. (at which wavelength 2-methyl-1:4-naphthoquinone absorbs maximally). 0·1 ml. ceric sulphate solution and 1 ml. cyclohexane were now added and the whole well shaken. The organic phase was seperated off and had an optical density of 0·412 at 250 m$\mu$. Since the molar extinctions of Synkavit at 290 m$\mu$. is 5600 and of the quinone at 250 m$\mu$. is 19,000 this simple extraction represents a recovery of about 79 per cent. Similar estimations are possible using bromine water as the oxidiser, but in this case excess bromine must be removed by a stream of air or nitrogen before extracting with cyclohexane.

*Biological experiments.*—The experimental mice were divided randomly into two groups. The " treated " mice were injected with 2 mg. Synkavit in 0·1 ml. saline and the " controls " with 0·1 ml. saline. Each mouse was injected with 0·1 ml. of the solution of labelled formate or glycine containing 1 mg. of the precursor and usually about 20 $\mu$c. The animals were sacrificed two hours later by breaking their necks.

*The acid soluble fraction.*—The ascites fluid, at least 8–10 ml. in each batch, was removed by a syringe into an ice cooled centrifuge tube containing a few drops of 2 per cent oxalate solution. An equal volume of cold 0·6 N $HClO_4$ was added and the mixture homogenised in an ice-cooled container (M.S.E. " Atomix " maximum speed for 1 minute). The homogenate and vessel washings were spun at 2000 r.p.m. for 5 minutes (M.S.E. Major centrifuge using precooled containers). The supernatant was filtered into an ice cooled Buchner flask through a pad of Hyflo over a sintered disc which had been well washed with ice water and then ice cold 0·6 N $HClO_4$. The tissue residue was suspended in 5–10 ml.

cold perchloric acid and spun down, the supernatant being filtered as before. This whole process was repeated. The filter pad was finally washed with about 5 ml. cold 0·6 N $HClO_4$. The volume of the acid soluble extract was measured and the solution clarified if necessary by filtering through a paper. A portion was diluted 1/5 with N/10 HCl and the optical density at 260 m$\mu$. (peak) measured. The separation of the purines from this fraction was usually done by evaporation and acid hydrolysis (Marshak and Vogel, 1951) but a method which gives cleaner separation on paper is as follows. The extract was neutralised with aqueous sodium hydroxide solution and 10 ml. 10 per cent silver nitrate added. The precipitate was spun off and heated at 100° with 10 ml. N HCl for 1 hour. The filtered hydrolysate was freed from acid by repeated evaporation *in vacuo* and addition of water, and the final residue dissolved in a little water and run on paper (Whatman 3 mm.) in iso-propanol/HCl (Wyatt, 1951). 10 ml. (about 1/8 of the total) of the neutralised acid soluble fractions (both treated and controls) was acidified with a few drops of concentrated sulphuric acid and gently homogenised with small volumes (1–4 ml.) of pure cyclohexane. The organic phase was sucked off, clarified by spinning, and the optical density at 250 m$\mu$. measured. This extraction was repeated until the cyclohexane had zero optical density : 3 and 6 per cent of the ultraviolet absorbing material was extracted from the control and treated solutions respectively under these conditions. Addition of ceric sulphate solution (or bromine water) caused a further 0·4 and 0·6 per cent to become organic soluble ; thus, the injected Synkavit could not have accounted for more than 0·6 per cent of the optical density of the treated acid soluble fraction, but it should be noted that the spectrum of this extracted material did not resemble 2-methyl-1:4-naphthoquinone. Aliquots of the treated and control ASF were diluted with N/10 HCl and the optical density at 260 m$\mu$ measured before and after treatment with excess bromine water, the excess being removed by aeration. The optical density of the treated ASF decreased from 7·10 to 4·90, 5·00 and that of the control from 7·15 to 5·68, 5·53 (duplicate estimations).

*The tissue residue*

The following manipulations were all carried out in the same weighed 6 in. B19 test tube to avoid losses. The tissue residue insoluble in cold dilute perchloric acid, including the small amount from the top of the Hyflo filter pad, was suspended twice in water, enough aqueous sodium hydroxide solution being added to the second suspension to bring the supernatant to neutrality. The insolubles were washed twice with alcohol, once with ether and refluxed for 1 hour with 20 ml. methanol/chloroform (1:1). The solids were then washed with alcohol, ether and dried *in vacuo* and the total weight noted. The dried solids were well mixed with a glass rod to ensure homogeneity.

*Separation of RNA and DNA purines* and counting of the samples was carried out as previously described (Marrian, 1954 ; Marrian, Hughes and Werba, 1956).

*Estimations of DNA* were carried out in duplicate on 20–30 mg. samples of the tissue residue as described by Burton (1956).

### RESULTS AND DISCUSSION

The results of these experiments are given in Tables I, II and III as the ratio between the specific activity of the purines in the Synkavit treated and in the control mice (Relative Specific Activity). The variability of the results necessitated many repetitions and, indeed, the final figures are the means of 22 experiments where formate was the precursor ; the addition of 5 experiments where glycine was used did not alter the figures appreciably. Occasionally, some of the results were so widely different from the means that some error either in manipulation or in the metabolic state of either the control or treated mice had occurred. Such results were eliminated from the final calculations by Chauvenet's criterion (Palmer, 1912, quoted and discussed by Calvin *et al.*, 1949) ; the number of results deleted and their value are given in the tables. The arithmetical means and standard deviations are given, and the means have been recalculated using logarithmic values since biological experiments are frequently better so expressed. In the present series however, there is no significant difference between the results by either calculation, but the logarithmic calculations require fewer deletions by Chauvenet's criterion. The results left little doubt that one effect of the injected Synkavit was to inhibit the synthesis of RNA purines from low molecular weight precursors without affecting the synthesis of either DNA purines or the acid soluble purine nucleotides. This suggested that the mode of action was to interfere with the polymerisation of the acid soluble nucleotides to RNA and it would follow that an accumulation of acid soluble nucleotides should occur. This was indeed shown to be the case by comparing the amount of acid soluble ultraviolet absorbing material per unit amount of DNA in the corresponding dry tissues residues in each case. The results of an experiment where 8 mice were divided randomly and treated as before is given in Table IV. The increase shown in Table IV in the amount of acid soluble nucleotides per unit amount of DNA in the Synkavit treated mice compared to the controls is not only in agreement with the postulated block between the acid soluble fraction and the RNA but the extent of the increase (23 per cent) is in very close agreement with the amount by which the activity of the treated RNA purines have fallen below the control RNA purines in the amount of precursor utilised (24 and 20 per cent for adenine and guanine respectively).

TABLE I.—*Acid Soluble Purines—Relative Specific Activities* (*R.S.A.*)

22 *Experiments with Formate as Precursor*

| | Number of observations | Observations deleted | R.S.A. Arithmetical | R.S.A. Logarithmic |
|---|---|---|---|---|
| Adenine | 22 | Ar. 0·34<br>Lg. 0·34 | 1·01±0·06 | 0·97 {+0·06, −0·04} |
| Guanine | 18 | Ar. 1·75, 1·94, 6·30<br>Lg. 6·30 | 1·09±0·07 | 1·12 {+0·11, −0·10} |

22 *Experiments with Formate and* 5 *with Glycine as Precursor*

| | Number of observations | Observations deleted | R.S.A. Arithmetical | R.S.A. Logarithmic |
|---|---|---|---|---|
| Adenine | 27 | Ar. 0·34, 0·29<br>Lg. 0·34, 0·29 | 0·99±0·05 | 0·95±0·05 |
| Guanine | 22 | Ar. 1·75, 1·94, 6·30<br>Lg. 6·30 | 1·05±0·07 | 1·07 {+0·08, −0·07} |

TABLE II.—*Deoxyribonucleic Acid Purines—Relative Specific Activities (R.S.A.)*

*22 Experiments with Formate as Precursor*

| | Number of observations | Observations deleted | R.S.A. Arithmetical | R.S.A. Logarithmic |
|---|---|---|---|---|
| Adenine | 21 | Ar. 3·6, 1·64, 2·52, 1·91<br>Lg. 3·6 | 0·90±0·06 | $0\cdot98\left\{^{+0\cdot11}_{-0\cdot08}\right.$ |
| Guanine | 21 | None | 1·00±0·06 | $0\cdot95\left\{^{+0\cdot05}_{-0\cdot07}\right.$ |

*22 Experiments with Formate and 5 with Glycine as Precursor*

| | Number of observations | Observations deleted | R.S.A. Arithmetical | R.S.A. Logarithmic |
|---|---|---|---|---|
| Adenine | 24 | Ar. 3·6, 1·64, 2·52, 1·91<br>Lg. 3·6 | 0·87±0·05 | $0\cdot93\left\{^{+0\cdot11}_{-0\cdot07}\right.$ |
| Guanine | 24 | None | 0·98±0·06 | $0\cdot94\left\{^{+0\cdot05}_{-0\cdot06}\right.$ |

TABLE III.—*Ribonucleic Acid Purines—Relative Specific Activities (R.S.A.)*

*22 Experiments with Formate as Precursor*

| | Number of observations | Observations deleted | R.S.A. Arithmetical | R.S.A. Logarithmic |
|---|---|---|---|---|
| Adenine | 20 | Ar. 5·0, 1·8, 6·3, 1·8<br>Lg. 5·0, 6·3 | 0·74±0·07 | $0\cdot74\left\{^{+0\cdot09}_{-0\cdot08}\right.$ |
| Guanine | 20 | Ar. 1·43, 1·78<br>Lg. 1·78 | 0·81±0·06 | 0·83±0·07 |

*22 Experiments with Formate and 5 with Glycine as percursor*

| | Number of observations | Observations deleted | R.S.A. Arithmetical | R.S.A. Logarithmic |
|---|---|---|---|---|
| Adenine | 25 | Ar. 5·0, 1·8, 6·3, 1·8<br>Lg. 5·0, 6·3 | 0·76±0·06 | 0·76±0·07 |
| Guanine | 25 | Ar. 1·43, 1·78<br>Lg. 1·78, 0·19 | 0·80±0·06 | 0·78±0·06 |

TABLE IV.—*Accumulation of Acid Soluble Nucleotides after Synkavit Treatment.*

| | Treated | Controls |
|---|---|---|
| Volume of ascites fluid | 20 ml. | 25 ml. |
| Total ASF by O.D. at 260 mμ. | 596 units | 604 units |
| Total DNA in the tissue residues by O.D. at 600 mμ. (Duplicate estimation) | 250, 252 units | 296, 292 units |
| Ratio DNA/ASF | 2·38 | 2·06 |
| Ratios from 3 earlier experiments | 2·0 | 1·6 |
| | 1·8 | 1·7 |
| | 2·1 | 1·5 |
| Mean | 2·1±0·1 | 1·7±0·1 |

It was, of course, necessary to show that the increase in acid soluble ultraviolet absorbing material was not due merely to the presence of the injected Synkavit or some water soluble derivative. However, ceric sulphate or bromine water estimations for the presence of Synkavit showed that the amount of Synkavit which could have been present was insignificantly small. The action of dilute bromine water on mixed purine and pyrimidine derivatives is to make the latter transparent to ultraviolet light, thus allowing a crude differential analysis. The ratio of pyrimidine to purines thus found in the control ASF was 0·28, while that in the treated was 0·44 suggesting that the increase in the nucleotides was not uniform, the pyrimidine nucleotides increasing in concentration relatively more than the purines.

The action of Synkavit as a radiosensitizer may therefore involve interference with RNA synthesis, while the X-ray is interfering with DNA synthesis, and this two-pronged attack on the cell's functions may account for the more than additive effect of combined treatments.

Some avenues for future work are suggested by these results. It will be interesting to see whether Synkavit will inhibit polynucleotide phosphorylase, the enzyme which Grunberg-Monago, Ortiz and Ochoa (1955) have shown to polymerise ribonucleoside diphosphates to RNA like material. Furthermore, if it is the two sided approach of combination therapy which is involved, then Synkavit may increase the desired effects of therapy by alkylating agents allowing, perhaps, remissions of acute leukaemia with less toxic side effects. And Actinomycin D, which appears to sensitize human skin to irradiation (Kingsley-Pillers—personal observations) may show this effect through an interference with RNA synthesis.

### SUMMARY

The effect of tetrasodium 2-methyl-1:4-naphthohydroquinone diphosphate (Synkavit) on the uptake of labelled formate or glycine into the purines of mouse ascites tumour has been studied over a 2-hour period. The compound does not alter the uptake of the precursor into the purines of the acid soluble nucleotides or of the deoxyribonucleic acid, but the uptake into the ribonucleic acid purines is reduced by 20–25 per cent compared to the controls. This inhibition is accompanied by an increase in 23 per cent of the total ultraviolet absorption of the acid soluble fraction which could not be accounted for by the presence of the injected compound in this fraction. The pyrimidines in this fraction increased in concentration relative to the purines.

The implications of these observations are discussed.

I gratefully record my thanks to Mrs. J. E. Hart and Miss J. Haird who assisted in these experiments, and to Mrs. I. Simon-Reuss and Mr. E. A. King who supplied me with the mice carrying the ascites tumour. The Synkavit used in these and many other experiments was generously given by Roche Products Limited, Welwyn Garden City, Herts. The automatic counter was purchased by means of a grant from the British Empire Cancer Campaign.

### REFERENCES

Burton, K.—(1956) *Biochem, J.*, **62**, 315.

Calvin, M., Heidelberger, C., Reid, J. C., Tolbert, B. M. and Yankwich, P. F.—(1949) " Isotopic Carbon ", New York (John Wiley and Sons Inc.), p. 290.

Clark, V. M., Kirby, G. W. and Todd, A. R.—(1958) *Nature*, **181**, 1650.

Cohen, L. and Cohen, A.—(1959) *Brit. J. Radiol.*, **32**, 18.

DITTRICH, W. AND SCHMERMUND, H. J.—(1953) *Strahlentherapie*, **90**, 88.
FREEDLANDER, B. L., REICH, S., LEVITAN, J. AND FRENCH, A.—(1958) *Cancer Res.*, **18** No. 8, Pt. 2), 447.
FRIEDMANN, E. AND BAILLEY, N. J. T.—(1950) *Biochim. biophys. Acta*, **6**, 274.
GELLHORN, A. AND GAGLIANO. T.—(1950) *Brit. J. Cancer*, **4**, 103.
GRUNBERG-MANAGO, M., ORTIZ, P. J. AND OCHOA, S.—(1955) *Science*, **122**, 907.
JOLLES, B.—(1952) *Rep. Brit. Emp. Cancer Campgn*, **30**, 324.
KLEIN, E. AND FORSSBERG, A.—(1954) *Exp. Cell. Res.*, **6**, 211.
MARRIAN, D. H.—(1954) *Biochim. biophys. Acta*, **13**, 282.
*Idem*, HUGHES, A. F. W. AND WERBA, S. M.—(1956) *Ibid.*, **19**, 318.
MARSHAK, A. AND VOGEL, H. J.—(1951) *J. biol. Chem.*, **189**, 597.
McKELVIE, A. D.—(1959) *Nature*, **183**, 1194.
MITCHELL, J. S.—(1952) *Rep. Brit. Emp. Cancer Campgn*, **30**, 238.—(1953) *Acta Radiol., Stockh.* (Congress Volume) Suppl. **116**, 431.—(1955) "Radiobiology Symposium" (Liege) 1954. London. (Butterworths Scientific Publications) p. 170.—(1959) *Brit. J. Radiol.*, **32**, 214.
*Idem* AND SIMON-REUSS, I.—(1947) *Nature*, **160**, 98.—(1952*a*) *Brit. J. Cancer*, **6**, 305.—(1952*b*) *Ibid.*, **6**, 317.
*Iidem* AND KING, E. A.—(1959) *Acta Un. int. Cancr.* (In press.)
PALMER, A. DE F.—(1912) "Theory of Measurements" New York., (McGraw Hill Book Co.), p. 127 *et seq.*
TARNOWSKI, G. S., BANE, H. N., CONRAD, J., NICKSON, J. J., STOCK, C. C. AND SUGIURA, K.—(1958) *Cancer Res.*, **18**, (No. 8, Part 2), 225.
WYATT, G. R.—(1951) *Biochem. J.*, **48**, 584.
YAMAGISHI, M.—(1954) *Rep. Takeda res. Lab.*, **13**, 25.